# Supplementary material for: The Selectively Nontargeted Analysis of Halogenated Disinfection Byproducts in Tap Water by Micro-LC QTOFMS
Source: Toxics. 2024 Aug 26;12(9):630. doi: 10.3390/toxics12090630 (PMC11436213; doi:10.3390/toxics12090630)
Supplement: Supplementary file 1 [file toxics-12-00630-s001.zip › toxics-3134459-supplementary.pdf]

Supplementary Material

The Selectively Nontargeted Analysis of Halogenated Disinfection Byproducts in Tap Water by Micro-LC QTOFMS

Jing Wu [1], Yulin Zhang [1], Qiwei Zhang [1], Fang Tan [1], Qiongyu Liu [2,*] and Xiaoqiu Yang [1,*]

[1] School of Optoelectronic Materials & Technology, Jianghan University, Wuhan 430056, China; 19503826580@163.com (J.W.); 18238778284@163.com (Y.Z.); keewezhang@jhun.edu.cn (Q.Z.); yanxizaof@sina.com (F.T.)
[2] College of Environment and Health, Jianghan University, Wuhan 430056, China
* Correspondence: qiongyuliu@163.com (Q.L.); xiaoqiuyang@jhun.edu.cn (X.Y.)

19 Pages

9 Texts

15 Figures

0 Table

Text S1. Solid phase enrichment. The pH of water samples was adjusted to 7~8 by adding NH_4OH before HLB enrichment, and to 2 with formic acid (FA) before Bond Elut ENV and Bond Elut C18 enrichment. Two hundred milliliter water samples went through each SPE cartridge under vacuum at a flow rate of 5 mL/min. Before elution, the cartridge was put into a Vacuum centrifugal concentrator (Concentrator plus, Eppendorf, German) with 30 ℃ for 30 min to dry. Organics enriched on HLB cartridges were eluted by 12 mL 85% acetonitrile (ACN), while the elution from Bond Elut ENV and Bond Elut C18 cartridges was performed with 12 mL 0.1% FA in 85% ACN. The extracts were concentrated through a nitrogen blowing concentrator (HGC-12A, Tianjin Hengao Technology Development Co., Ltd, China) with nitrogen flow to near dry. The residues were reconstructed with 0.5 mL 0.1% FA in H_2O or 0.5 mL 0.1% FA in ACN and MeOH (7:3, v/v).

Text S2. **Elucidation of ion cluster m/z 193.8864/195.9109/197.8108/199.8036.** According to the isotope characteristic of ion cluster 193.8864/195.9109/197.8108/199.8036, it was assumed that this compound had 4 Cl atoms. This ion cluster was even number, so the molecular of this compound should be odd number and contain one N atom. According to the molecular weight, the chemical formula was deduced to be C2HCl4NO. Put the chemical formula of C2HCl4NO in the search box of https://www.chemspider.com/, three candidate compounds were listed, and they were N,2,2,2-Tetrachloroacetamide, (1E)-2,2,2-Trichloro-N-hydroxyethanimidoyl chloride, and 1,1,1,2-Tetrachloro-2-nitrosoethane. (1E)-2,2,2-Trichloro-N-hydroxyethanimidoyl chloride is unstable in water and is easy to be hydrolyzed. 1,1,1,2-Tetrachloro-2-nitrosoethane is not easy to form [M-H]- in the negative mode of ESI ion source. Therefore, N,2,2,2-Tetrachloroacetamide was chosen and was assumed as a new DBP comparing with the DBP database and the latest DBP literatures. The MS spectra and proposed structure were demonstrated in Fig. S8.

Text S3. Elucidation of ion cluster *m/z* 190.9587/192.9563. According to isotopic characteristic in the TOFMS spectra at RT of 14.7 min, it was assumed that this compound contained one Br atom. The chemical formula was calculated as $C_5H_5O_3Br$ based on the monoisotopic mass of 190.9587. Comparing with the DBP database, two DBPs ((Z)-3-bromo-4-oxopent-2-enoic acid[1] and (E)-2-bromo-3-methyl-4-oxobut-2-enoic acid[2]) with the same formula of $C_5H_5O_3Br$ were proposed. The MS/MS spectra of (Z)-3-bromo-4-oxopent-2-enoic acid was demonstrated in the literature[1], and the fragment ion of 146.9451 was corresponding to 147.0470 obtained in this paper (Fig. S9)，while the fragment ion of 113.0230 was detected in this work. Therefore, this compound could not definitely identified as (Z)-3-bromo-4-oxopent-2-enoic acid, it could be (E)-2-bromo-3-methyl-4-oxobut-2-enoic acid or the other isomers。

Text S4. Elucidation of ion cluster *m/z* 229.1074/231.0884. According to the TOFMS spectra at 15.21 min and the monoisotopic mass of 229.1074, the chemical formula of this compound was calculated as $C_{10}H_{11}ClO_4$. From MS/MS spectra, the fragment ion of 193.0500 might be $[M\text{-}H\text{-}HCl]^-$（the difference between 193.0500 and 229.1074 was 36.0574， which is the mass of HCl. The fragment of 193.0500 continued be decarboxylated to form 149.0595, and 149.0595 lost a methyl and became the fragments of 134.0364. Additionally, the fragment of 178.0236 might be a product of 193.0500 losing a methyl. Based on the above elucidation, this compound was proposed

as 2-(5-Chloro-2-hydroxy-3- (hydroxymethyl)-4-methylphenyl) acetic acid, and the proposed structure of this DBP and fragments were demonstrated in Fig. S10. Input the formula of $C_{10}H_{11}ClO_4$ into the DBP database, and it was found that it was assumed as a DBP formed in chlorination combined UV [3] without specific structure.

Text S5. Elucidation of ion cluster *m/z* 267.1217/269.1199/271.1162. According to the isotope characteristic of TOFMS spectra at 17 min and the monoisotopic mass of 267.1217, the chemical formula of this compound was calculated as $C_7H_6ClBrO4$. From the MSMS spectra, it was seen that there were two continuous decarboxylation (267.1217 lost 44 to form the fragment of 223.1318, and 223.1318 continued to lose 44 forming the fragment of 179.1064). Therefore, this compound might have two carboxyl. Combing other fragments, it was speculated as Monobromochlorohexanedioic acid, the proposed structure and the formation of fragments were exhibition in Fig. S11.

Text S6. Elucidation of ion cluster *m/z* 224.9189/226.9145. According to the isotope characteristic of TOFMS spectra at 18 min and the monoisotopic mass of 267.1217, the chemical formula of this compound was calculated as $C_5H_7BrO_3S$. It is speculated as a compound with one carboxyl. Comparing the candidate compound list of $C_5H_7BrO_3S$ in the website of *Chemspider*, 3-[(2-Bromoethyl) sulfanyl]-2-oxopropanoic acid was more appropriate to meet the TOFMS and MS/MS spectra. The proposed structure of this compound and the formation of fragments were demonstrated in Fig. S12.

Text S7. Elucidation of ion cluster *m/z* 171.0206/173.0408. According to the isotope characteristic of TOFMS spectra at 18 min and the monoisotopic mass of 267.1217, the chemical formula of this compound was calculated as $C_7H_5ClO_3$. Comparing with the DBP database, there were three DBPs related to $C_7H_5ClO_3$, 5-Chlorosalicylic acid[4], 3-Chlorosalicylic acid[4], and one without specific structure [5]。 The fragment of 126.9955/128.9925 in MS/MS spectra of 171.0206/173.0408 formed by decarboxylation. Furthermore, there was no other available fragments for further structural elucidation, so the specific structure of $C_7H_5ClO_3$ could not be confirmed. It could be 5-Chlorosalicylic acid, 3-Chlorosalicylic acid, or their other isomers. The proposed structure and MS spectra were demonstrated in Fig. S13.

Text S8. Elucidation of ion cluster *m/z* 138.0385/140.0352. According to the isotope characteristic of TOFMS spectra at 21.2 min and the monoisotopic mass of 138.0385, the chemical formula of this compound was calculated as $C_4H_{10}ClNO_2$。 Inputting $C_4H_{10}ClNO_2$ into the DBP web database, the matched DBP was 1-aminoxy-1-chloro-butan-2-ol [6]. 1-aminoxy-1-chloro-butan-2-ol was found in 2001, its structure and EI, CI fragmental scheme were elucidated. While the MS/MS spectra of 138.0385/140.0352 detected in this work was different with that of 1-aminoxy-1-chloro-butan-2-ol. From Fig. S17, it could be seen the fragment of $[M-H-30]^-$and $[M-H-28]^-$. In the EI fragmentation procedure of 1-aminoxy-1-chloro-butan-2-ol, there were apparent fragment of m/z 107/108, which was a product losing NH_2O group. The mass loss of 28 detected in this work might be CH_2CH_2, and the loss of 30 might be OCH_2. Therefore, combing the MS information and the fragmentation

scheme propose in the literature [6], it was speculated as 1-(aminomethoxy)-1-chloropropan-1-ol. The proposed structure of this compound and its fragments were exhibited in Fig. S14.

Text S9. Elucidation of ion cluster *m/z* 286.0484/288.0526. According to the isotope characteristic of TOFMS spectra at 30.2 min and the monoisotopic mass of 286.0484, the chemical formula of this compound was calculated as $C_{12}H_{14}Cl\,NO_5$. The Double-bond equivalent (DBE) was 6, so it might contain a benzene ring (DBE = 4). The loss of 44 meant that this compound had a carboxyl group (DBE=1), and the loss of 30 indicated that it contained a nitro-group (DBE=1). Combining the other MS information this compound was speculated as 2-chloro-5-(3-(hydroxymethy)-5-nitrophenyl)pentanoic acid or the isomer of ortho, para in benzene ring. The proposed structure and its fragments were demonstrated in Fig. S15. Comparing with the DBP database and the latest literatures about DBPs, it was found this compound hasn't been reported, therefore, it could be assumed as a new DBP.

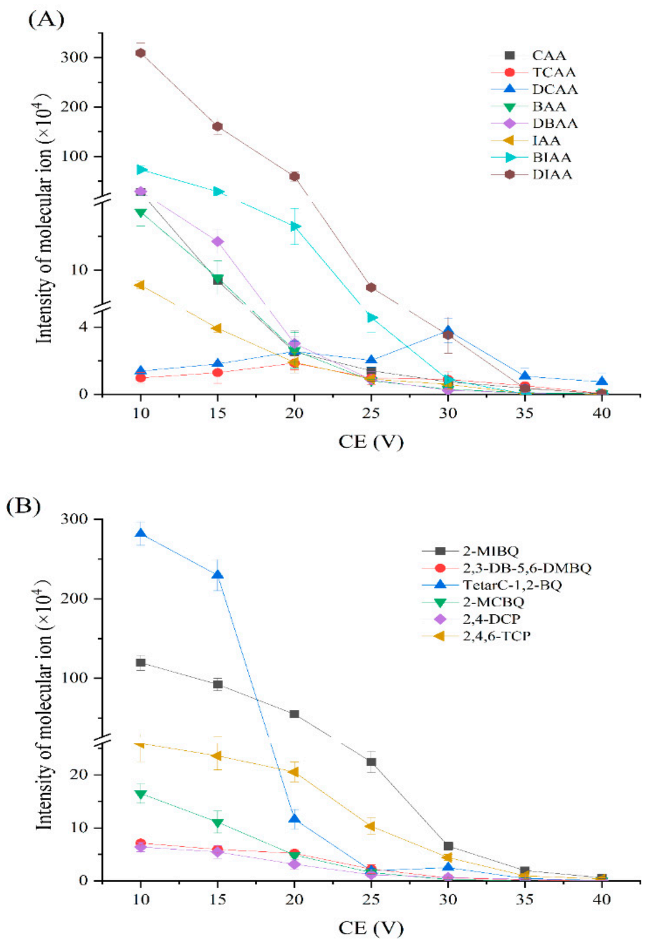

Figure S1. Intensity of molecular ion under various CE value in product ion mode (CAA: monochloroacetic acid, TCAA: trichloroacetic acid, DCAA: dichloroacetic acid, BAA: monobromoacetic acid, DBAA: dibromoacetic acid, IAA: monodiodoacetic acid, BIAA: bromodiodoacetic acid; 2-MIBQ: 2-monoiodobenzoquinone, 2,3-DB-5,6-DMBQ: 2,3-dibromo-

5,6-dimethylbenzoquinoen, Tetra C-1,2-BQ: tetrachloro-1,2-benzoquinone; 2-MCBQ: 2-monochlorobenzoquinone; 2,4-DCP: 2,4-dichlorophenol; 2,4,6-TCP: 2,4,6-trichlorophenol).

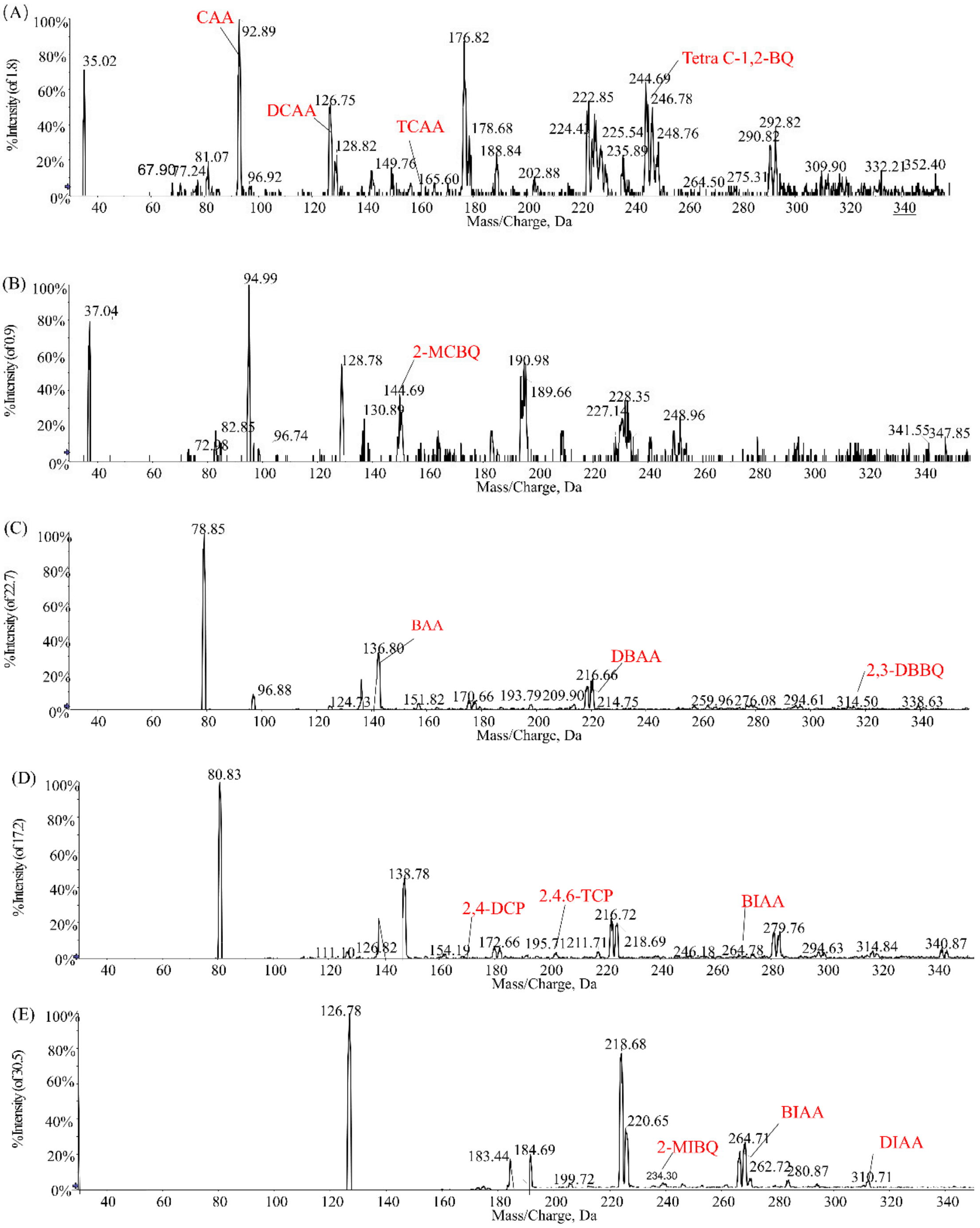

Figure S2. MS spectra of precursor ion of Cl^{35} (A), C^{l37} (B), Br^{79} (C), Br^{81}(D), I^{127}(E) under the CE value of 20 V

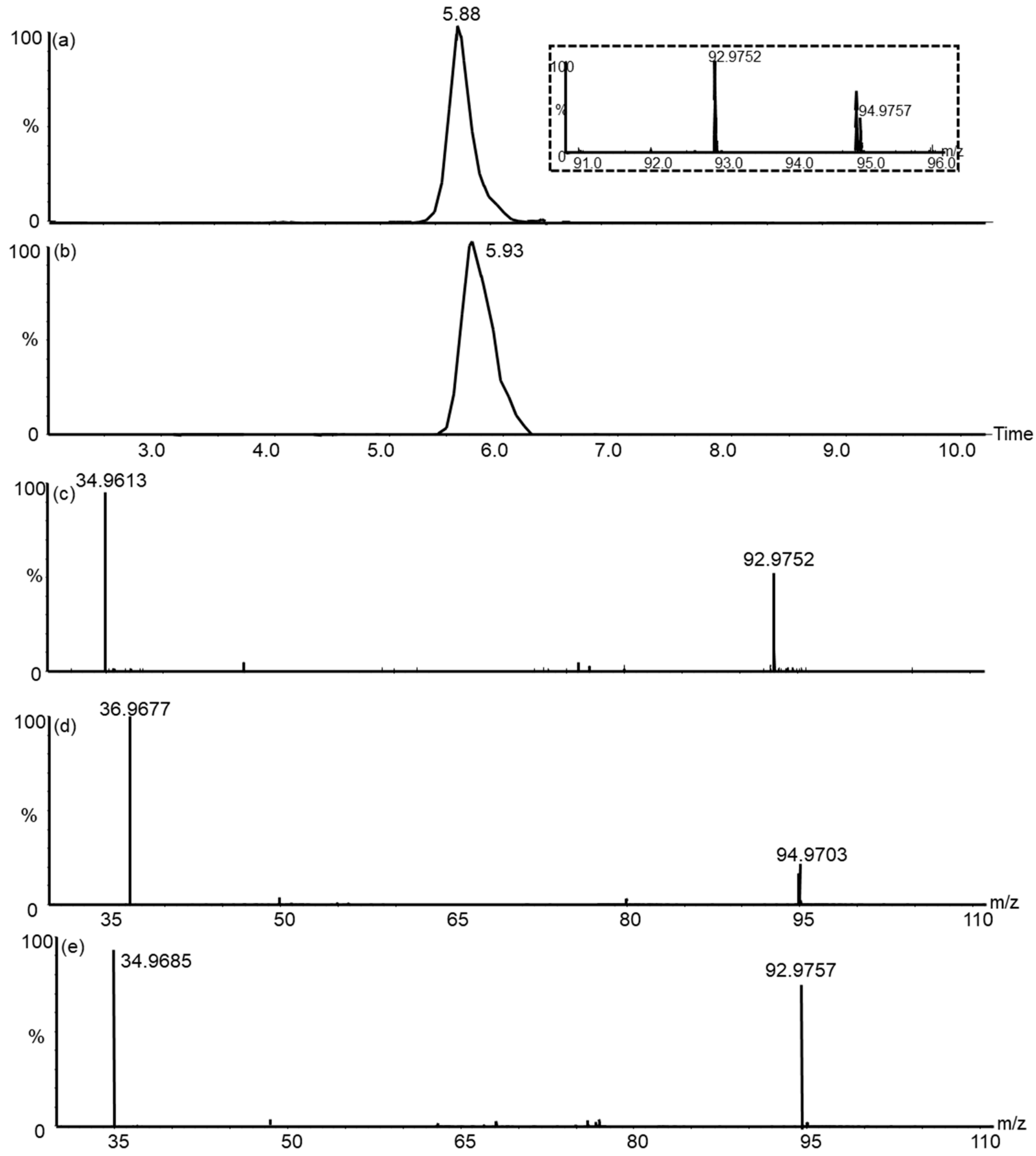

Figure S3. The conformation information of monochloroacetic acid. ((a) extraction ion chromatograph (EIC) of 34.9613 from product ion spectra (PIS) of 92.9 in HOCs solution; (b) EIC of 34.9613 from PIS of 92.9752 in T1 water sample; (c) and (d), MS/MS spectra in 5.9 min from PIS of 92.9752 and 94.9703 in T1 water sample, respectively; (e) MS/MS spectra in 5.9 min from PIS of 92.9752 in HOCs solution)

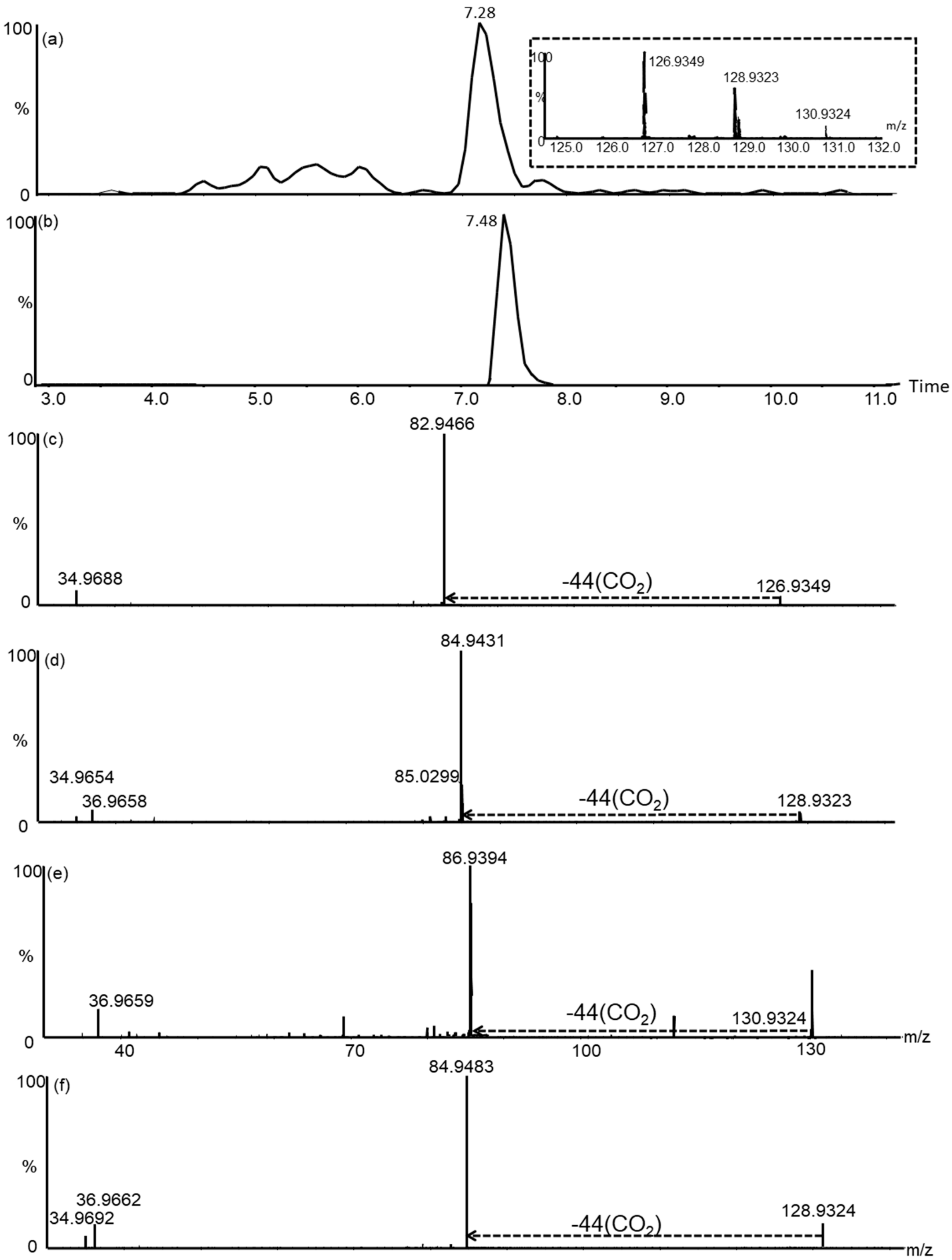

Figure S4. The conformation information of dichloroacetic acid ((a)-(b) EIC of 82.9466 from PIS of 126.9 in HOCs solution and T1 water sample; (c), (d), and (e) MS/MS spectra in 7.3 min from PIS of 126.99349, 128.9323 and 130.9324 in T1 water sample, respectively; (f) MS/MS spectra in 7.3 min from PIS of 128.9323 in HOCs solution).

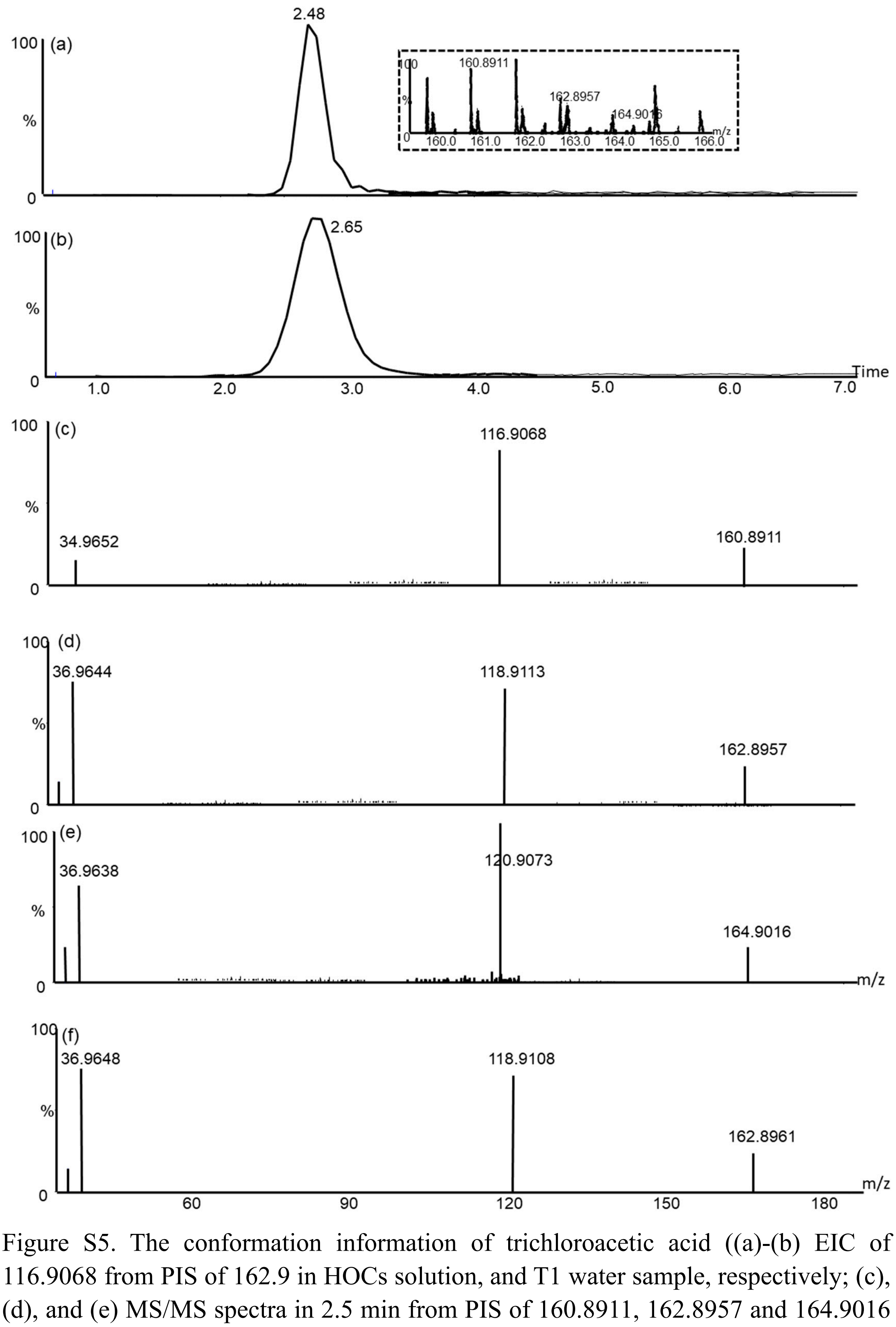

Figure S5. The conformation information of trichloroacetic acid ((a)-(b) EIC of 116.9068 from PIS of 162.9 in HOCs solution, and T1 water sample, respectively; (c), (d), and (e) MS/MS spectra in 2.5 min from PIS of 160.8911, 162.8957 and 164.9016 in T1 water sample, respectively; (f) MS/MS spectra in 2.5 min from PIS of 162.8957 in HOCs solution).

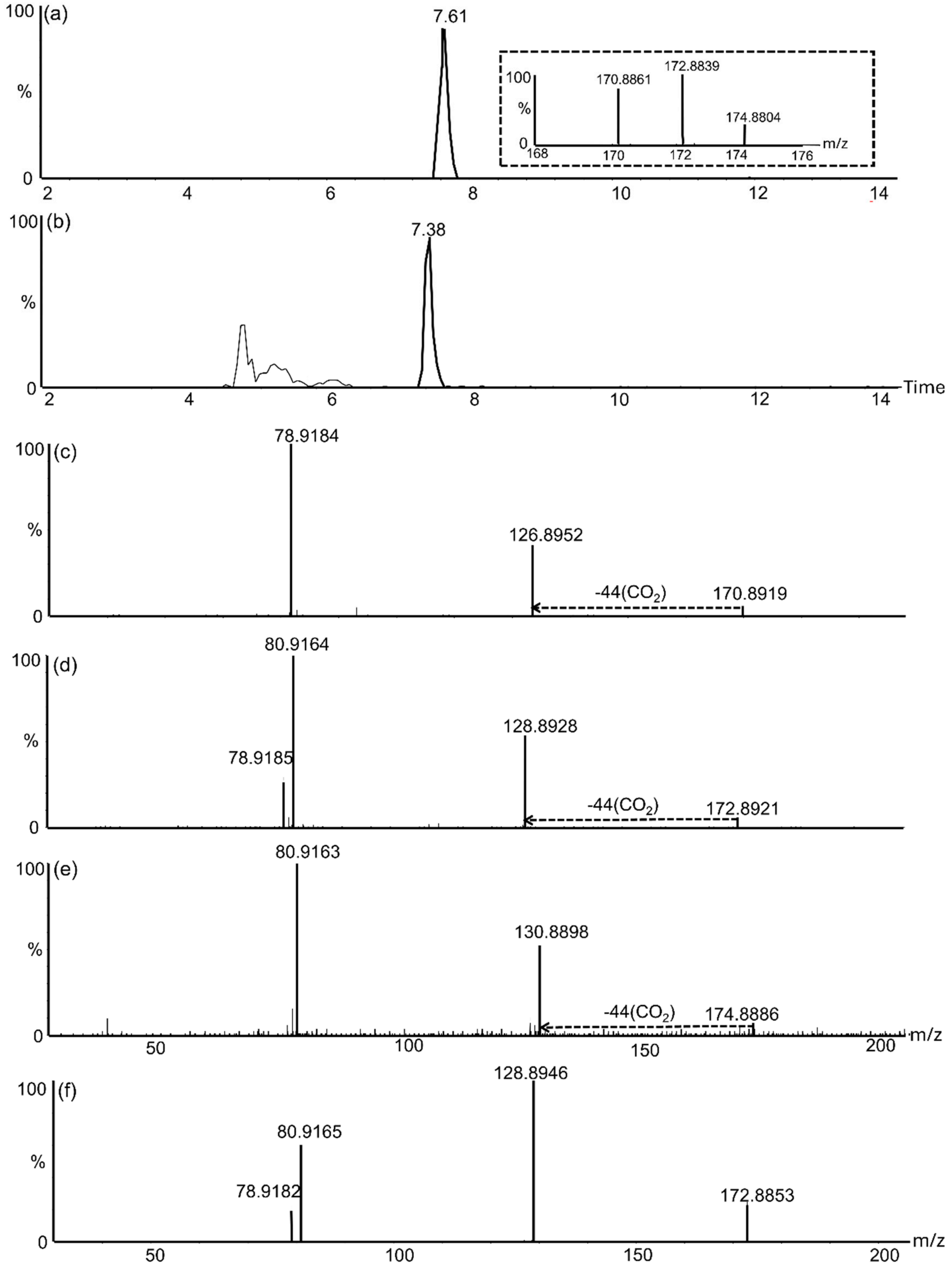

Figure S6. The conformation information of bromochloroacetic acid ((a)-(b) EIC of 126.8952 from PIC of 172.8921 in HOCs solution T1 water sample, respectively; (c), (d), and (e) MS/MS spectra in 2.5 min from PIS of 170.8919, 172.8921 and 174.8886 in T1 water sample, respectively; (f) MS/MS spectra in 7.5 min from PIC of 172.8921 in HOCs solution).

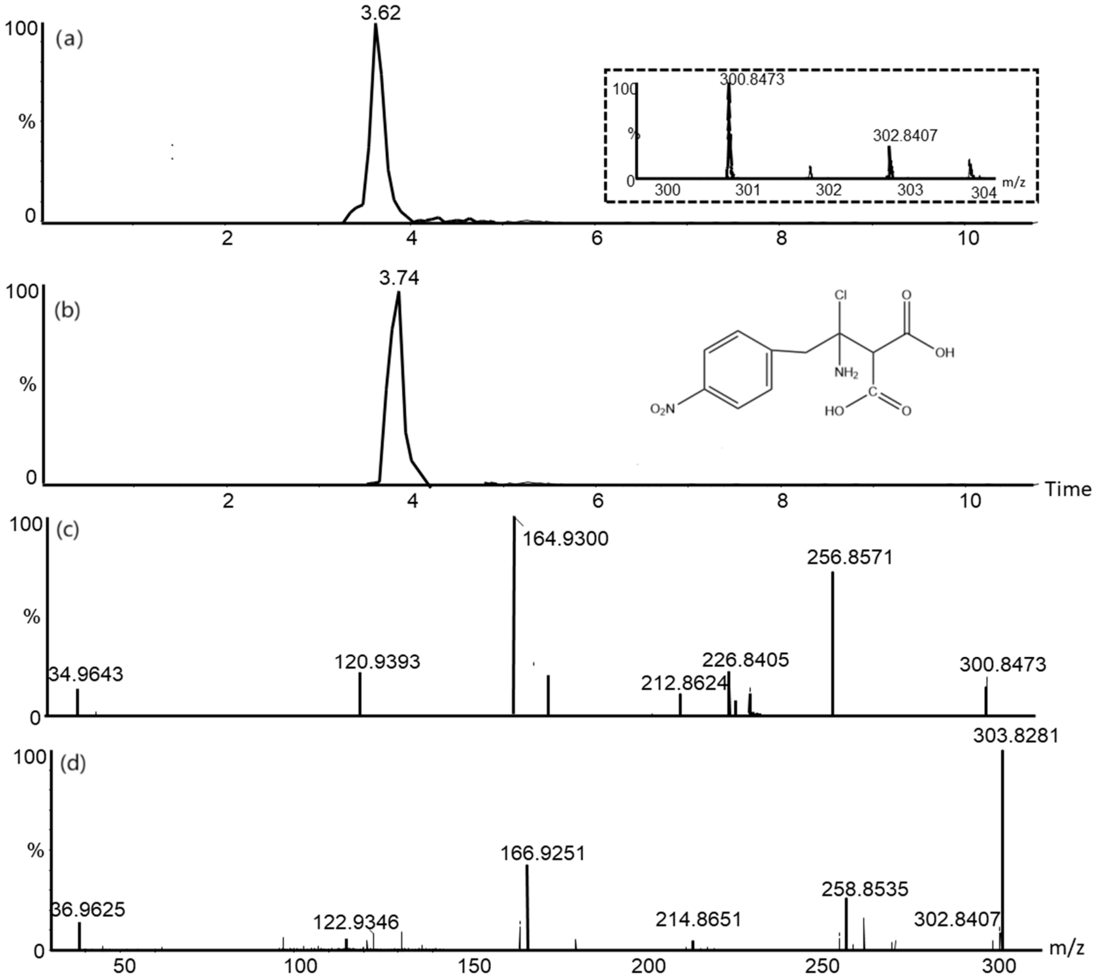

Figure S7. Compound deduction of the ion cluster m/z 300.8473/3002.8407 ((a) XIC of 300.8473 in TOF-MS chromatograph. (b) XIC of 302.8407 in TOF-MS chromatograph. (c) MS/MS spectra in 3.7 min from PIS of 300.8473. (d) MS/MS spectra in 3.7 min from PIS of 302.8407).

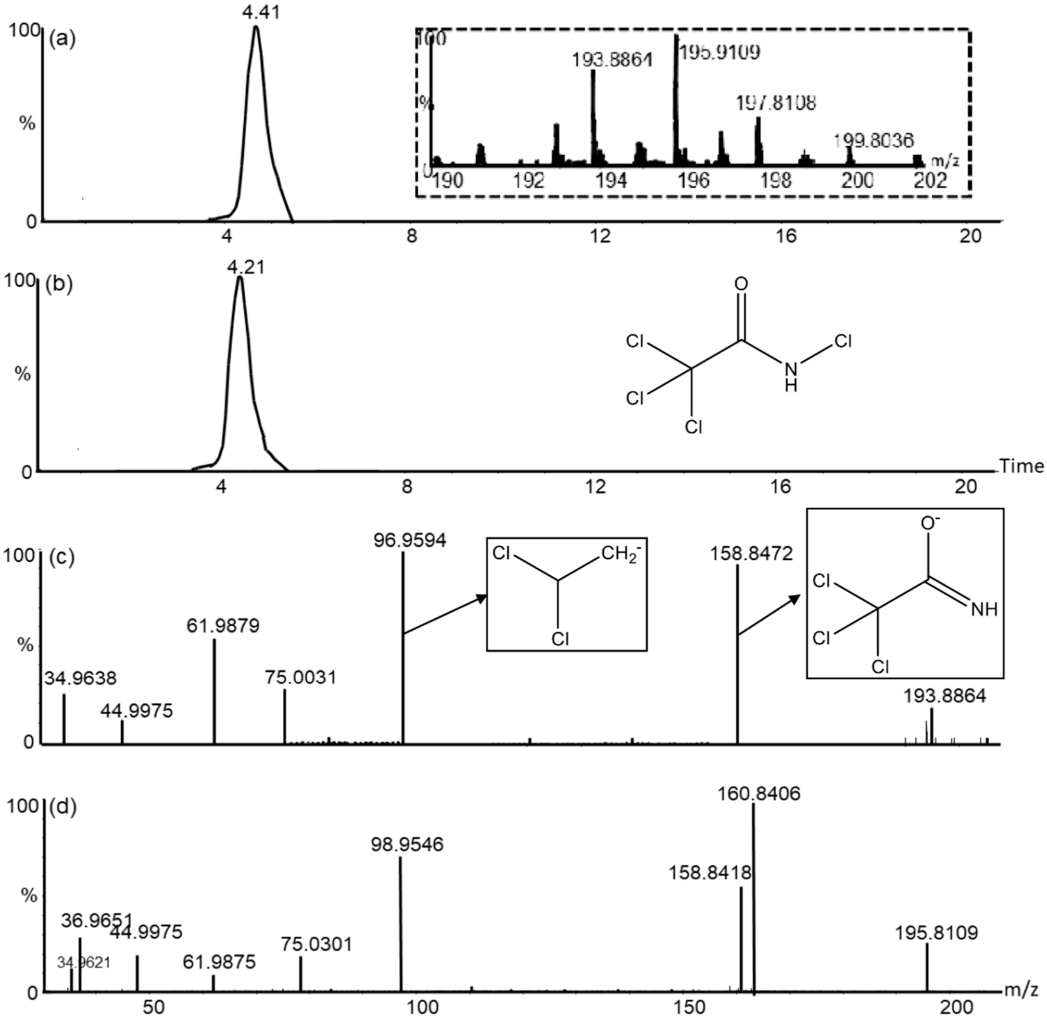

Figure S8. Compound deduction of the ion cluster 193.8864/195.9109/197.8108/199.8036 ((a) XIC of 93.8864 in TOFMS chromatograph. (b) XIC of 195.9109 in TOFMS chromatograph. (c) MS/MS spectra in 4.4 min from PIS of 193.8864. (d) MS/MS spectra in 4.4 min from PIS of 195.9109).

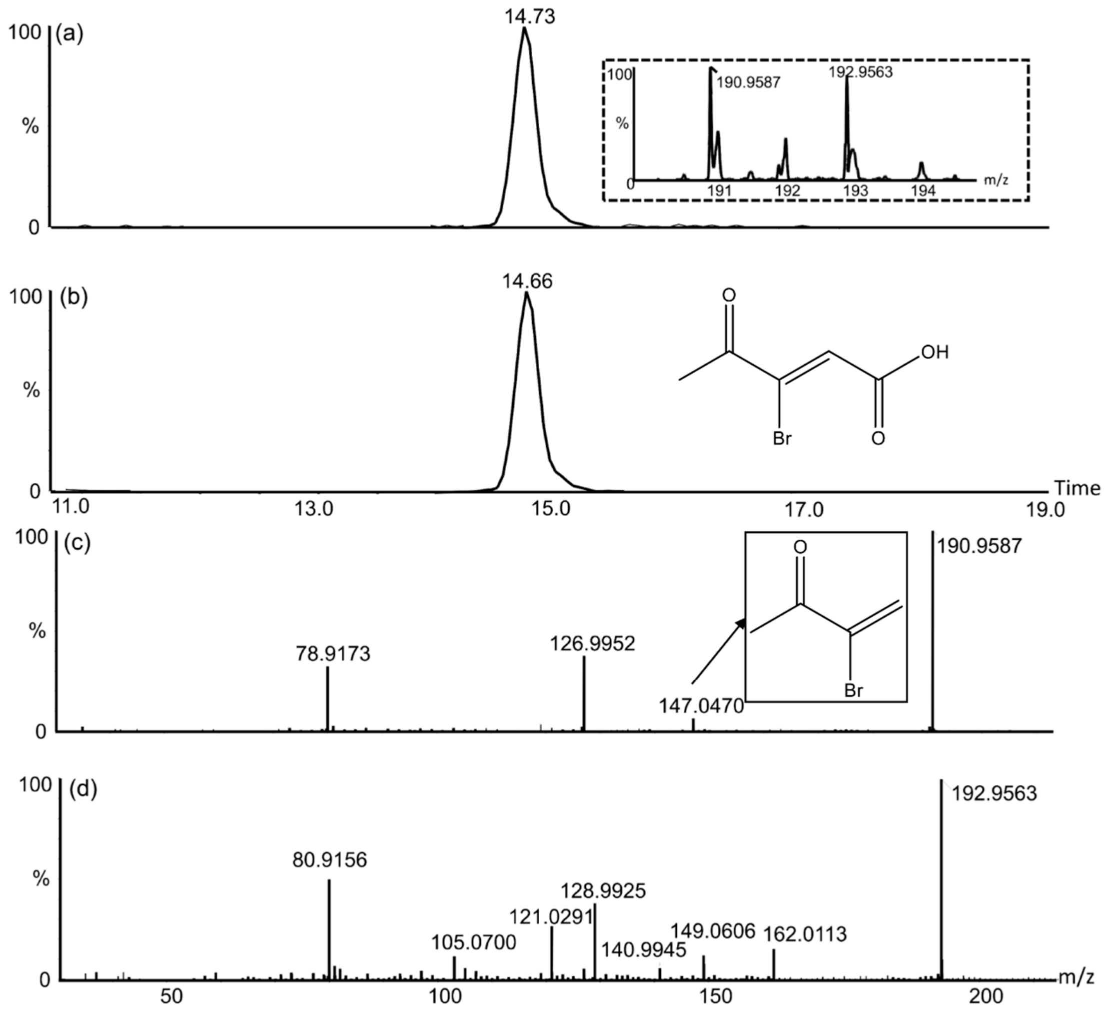

Figure S9. Compound deduction of the ion cluster 190.9587/192.9563 ((a) XIC of 190.9587 in TOFMS chromatograph. (b) XIC of 192.9563 in TOFMS chromatograph. (c) MS/MS spectra in 14.7 min from PIS of 190.9587. (d) MS/MS spectra in 14.7 min from PIS of 192.9563).

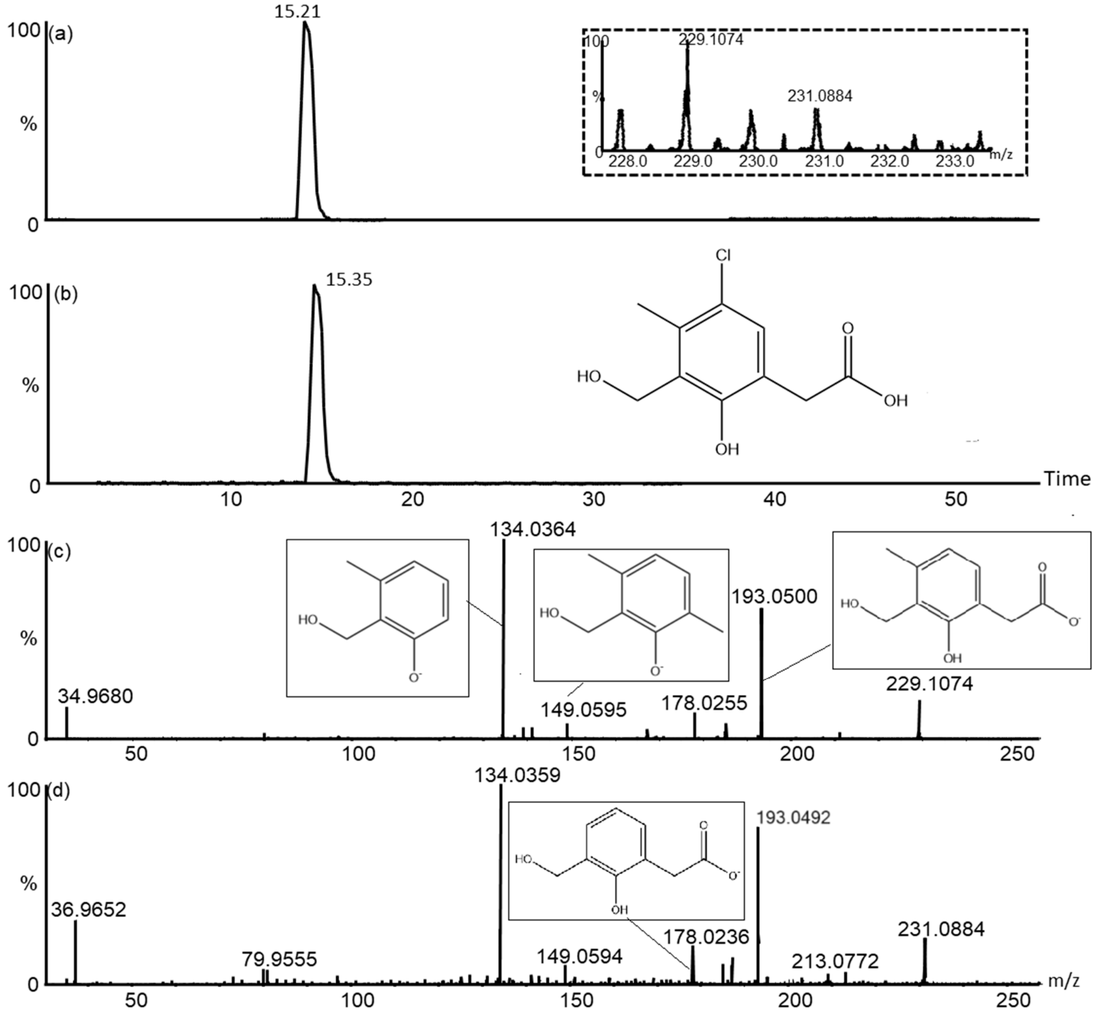

Figure S10. Compound deduction of ion cluster 229.1074/231.0884 ((a) XIC of 229.1074 in TOFMS chromatograph; (b) XIC of 231.0884 in TOFMS chromatograph and the proposed structure; (c) MS/MS spectra at 15.3 min from PIS of 229.1074 and the proposed structure of the fragments of 193.0500, 149.0595, and 134.0364; (d) MS/MS spectra at 15.3 min from PIS of231.0884 and the proposed structure of the fragment of 178.0236).

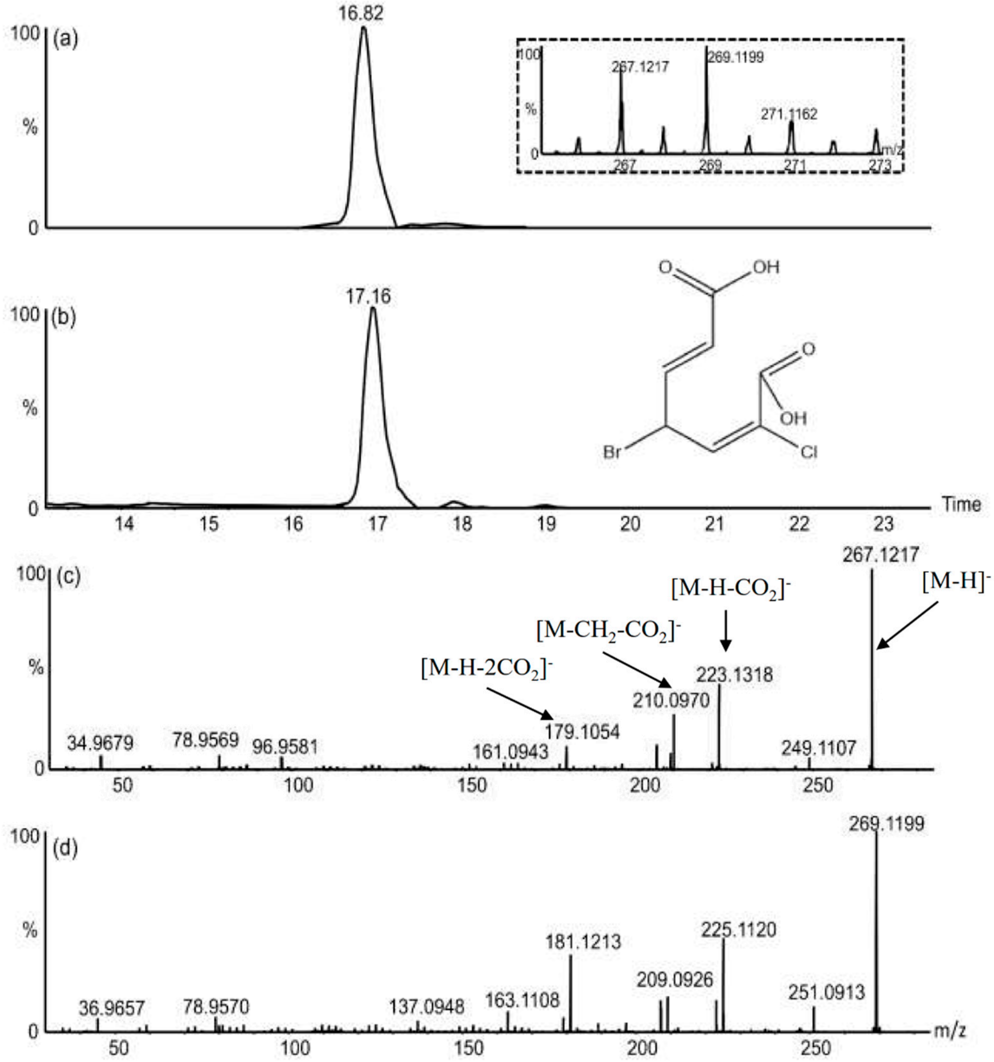

Figure S11. Compound deduction of ion cluster 267.1217/269.1199/271.1162 ((a) XIC of 267.1217 in TOFMS chromatograph; (b) XIC of 269.1199 in TOFMS chromatograph and the proposed structure; (c) MS/MS spectra at 17.1 min from PIS of 267.1217 (d) MS/MS spectra at 17.1 min from PIS of 269.1199).

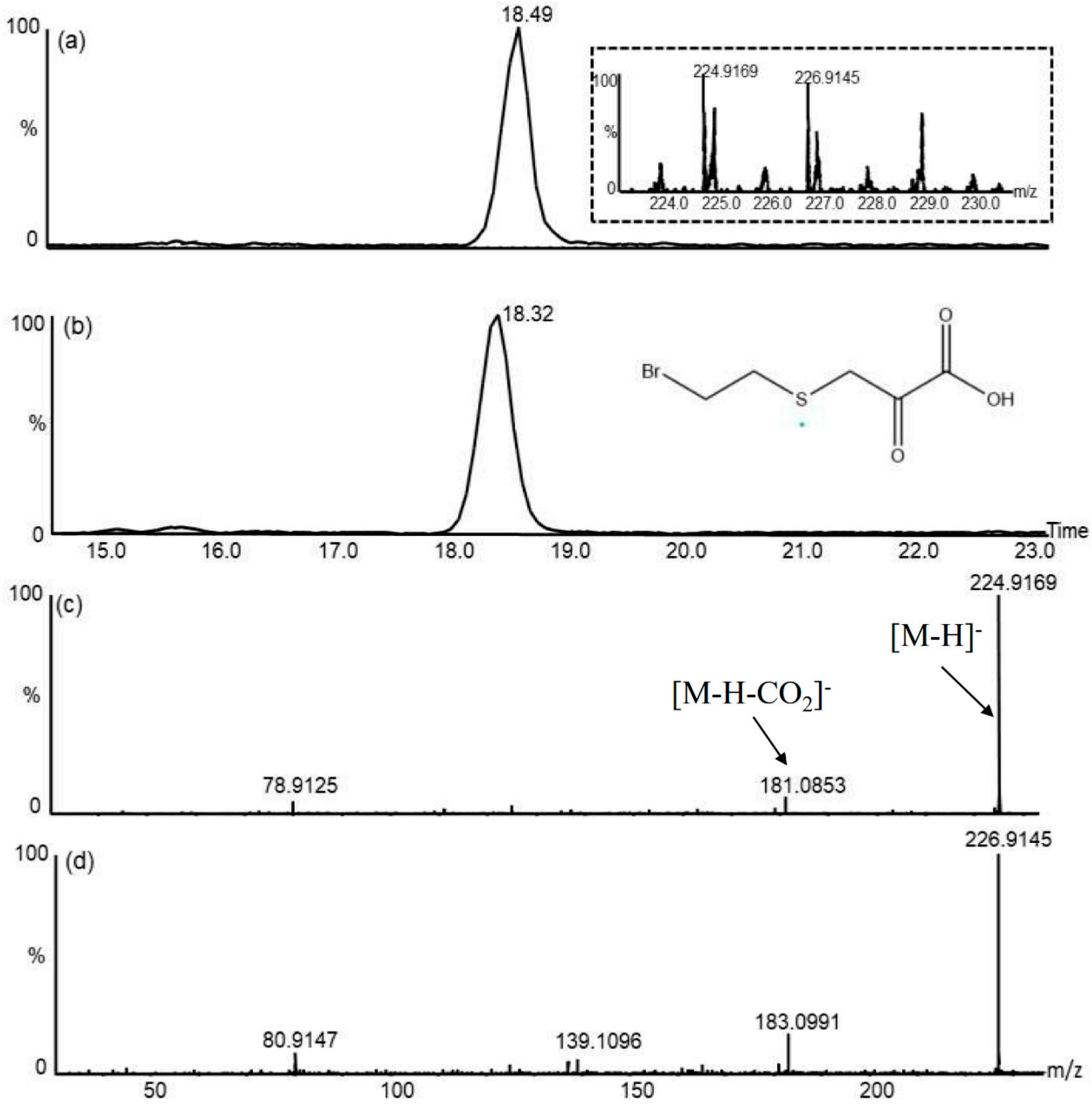

Figure S12. Compound deduction of ion cluster 224.9189/226.9145 ((a) XIC of 224.9189 in TOFMS chromatograph; (b) XIC of 226.9145 in TOFMS chromatograph and the proposed structure; (c) MS/MS spectra at 18.3 min from PIS of 224.9189 (d) MS/MS spectra at 18.3 min from PIS of 226.9145).

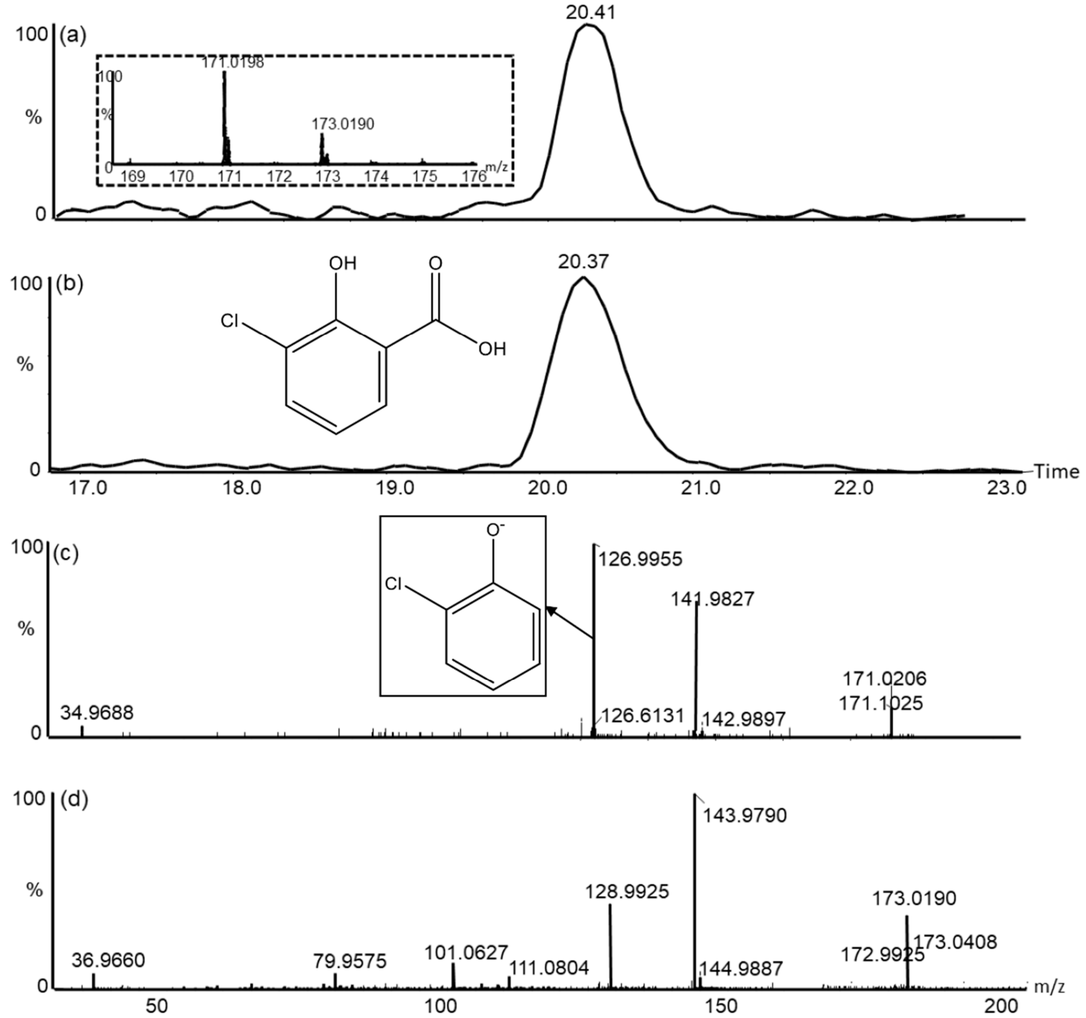

Figure S13. Compound deduction of ion cluster 171.0206/173.0408 ((a) XIC of 171.0206 in TOFMS chromatograph; (b) XIC of 173.0408 in TOFMS chromatograph and the proposed structure; (c) MS/MS spectra at 20.3 min from PIS of 171.0206 (d) MS/MS spectra at 20.3 min from PIS of 173.0408)

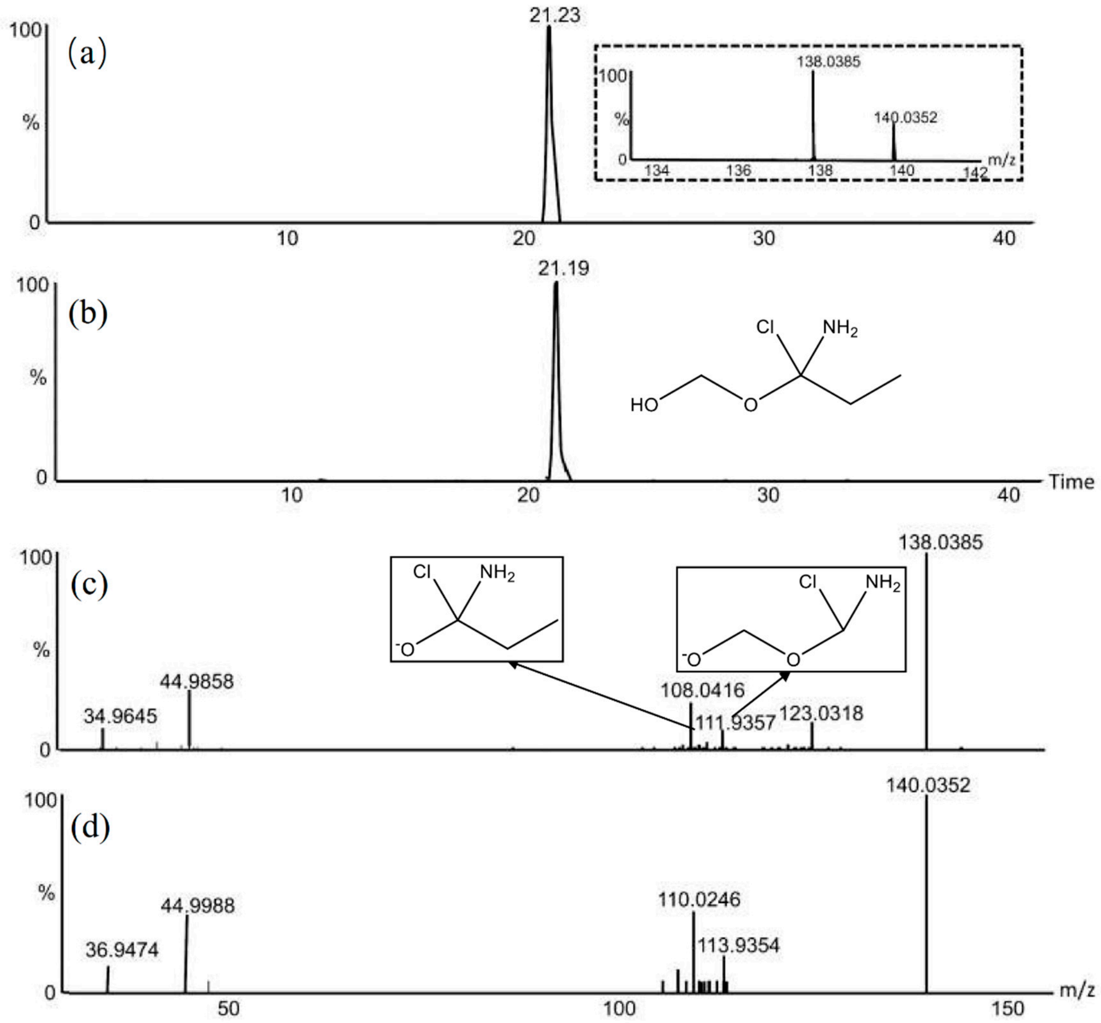

Figure S14. Compound deduction of ion cluster 138.0385/140.0352 ((a) XIC of 138.0385 in TOFMS chromatograph; (b) XIC of 140.0352 in TOFMS chromatograph and the proposed structure; (c) MS/MS spectra at 21.2 min from PIS of 138.0385 (d) MS/MS spectra at 21.2 min from PIS of 140.0352).

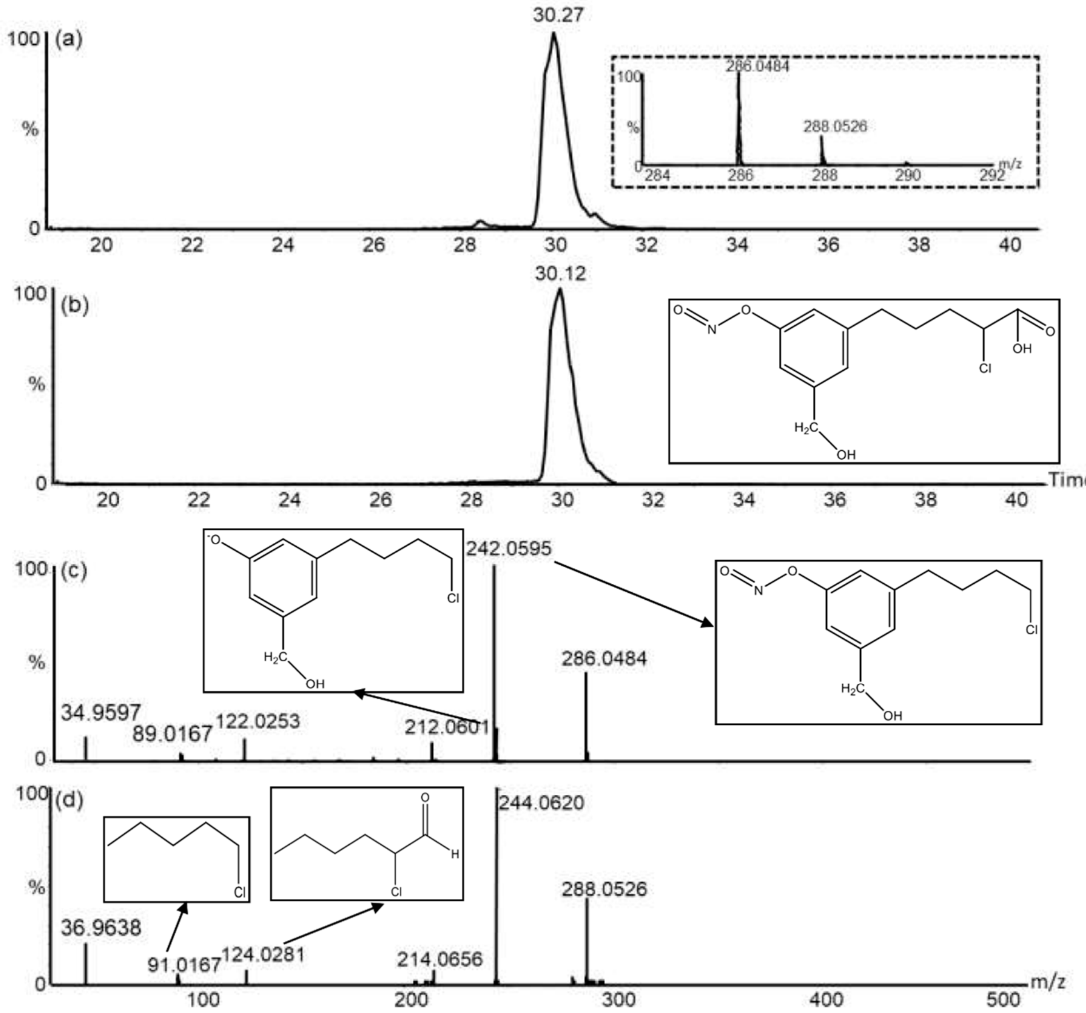

Figure S15. Compound deduction of ion cluster 286.0484/288.0526 ((a) XIC of 286.0484 in TOFMS chromatograph; (b) XIC of 288.0526 in TOFMS chromatograph and the proposed structure; (c) MS/MS spectra at 30.2 min from PIS of 286.0484 (d) MS/MS spectra at 30.2 min from PIS of 288.0526).

Reference

[1] Lu, Y.; Song, Z.-M.; Wang, C.; Liang, J.-K.; Hu, Q.; Wu, Q.-Y. Combining high resolution mass spectrometry with a halogen extraction code to characterize and identify brominated disinfection byproducts formed during ozonation. *Sci Total Environ*, 2021, *796*, 149016.

[2] Liu, X.; Liu, R.; Zhu, B.; Ruan, T.; Jiang, G. Characterization of Carbonyl Disinfection By-Products During Ozonation, Chlorination, and Chloramination of Dissolved Organic Matters. *Environ Sci Technol*, 2020, *54*, 2218-2227.

[3] Lavonen, E.E.; Gonsior, M.; Tranvik, L.J.; Schmitt-Kopplin, P.; Kohler, S.J. Selective Chlorination of Natural Organic Matter: Identification of Previously Unknown Disinfection Byproducts. *Environ Sci Technol*, 2013, *47*, 2264-2271.

[4] Bulloch, D.N.; Nelson, E.D.; Carr, S.A.; Wissman, C.R.; Armstrong, J.L.; Schlenk, D.;

Larive, C.K. Occurrence of Halogenated Transformation Products of Selected Pharmaceuticals and Personal Care Products in Secondary and Tertiary Treated Wastewaters from Southern California. *Environ Sci Technol*, 2015, *49*, 2044-2051.
[5] Postigo, C.; Andersson, A.; Harir, M.; Bastviken, D.; Gonsior, M.; Schmitt-Kopplin, P.; Gago-Ferrero, P.; Ahrens, L.; Ahrens, L.; Wiberg, K. Unraveling the chemodiversity of halogenated disinfection by-products formed during drinking water treatment using target and non-target screening tools. *J Hazard Mater*, 2021, *401*, 123681.
[6] Taguchi, V.Y. Structural elucidation of disinfection by-products in treated drinking water. *Rapid Commun Mass Spectrom*, 2001, *15*, 455-461.
